# 2009年北京胸外科学术年会隆重召开

**DOI:** 10.3779/j.issn.1009-3419.2010.01.16

**Published:** 2010-01-20

**Authors:** 修益 支

**Affiliations:** 100053 北京，首都医科大学肺癌诊疗中心，首都医科大学宣武医院胸外科 Beijing Lung Cancer Center & Thoracic Surgery Department, Xuanwu Hospital, Capital Medical University, Beijing 100053, China

2009年12月13日，2009年北京胸外科学术年会在北京裕龙国际酒店隆重召开。来自北京地区各系统近80家医院的近800名胸外科医生和护士长出席了首都胸外科领域的学术盛会，在京的胸外科老前辈徐乐天、李曰民、赵志文、孙玉鹗、李泽坚、王天佑、仇欣荪、张汝刚教授等几十位老教授出席了会议。来自上海、天津、河北、黑龙江、吉林、山西、广东和江苏等十几个省市胸心血管外科学会的几十位著名胸外科专家应邀出席了会议。

北京医学会胸外科专业委员会主任委员支修益教授、北京医学会会长金大鹏教授、中华医学会副秘书长杨民教授、中华医学会胸心血管外科学分会主任委员胡胜寿教授、中国医师协会胸外科医师分会会长王天佑教授、天津市胸心血管外科学会主任委员张逊教授和中国人民解放军空军总医院马中立院长分别在开幕式上发表了热情洋溢的讲话。北京医学会会长金大鹏教授、北京医学会胸外科专业委员会主任委员支修益教授为北京医学会胸外科专业委员会名誉主任委员徐乐天教授和王天佑教授颁发了“学会杰出贡献奖”。北京医学会会长金大鹏教授、中华医学会副秘书长杨民教授、中华医学会胸心血管外科学分会主任委员胡胜寿教授和北京医学会项晓培秘书长为李曰民教授、赵志文教授、张大为教授、孙玉鹗教授、张汝刚教授、董宗俊教授、李泽坚教授、陈鸿义教授、张志庸教授和臧德安教授颁发了获奖证书，以表彰他们为北京胸外科事业所做出的突出贡献。徐乐天教授和孙玉鹗教授代表获奖老专家即兴发言，对北京胸外科学会成立以来所做的各项工作给予了充分的肯定，对学会今后的工作提出了希望，希望团结北京地区胸外科学科带头人和广大胸外科医生，共同推进北京胸外科事业的进步与发展。

北京协和医院徐乐天教授、中国医学科学院肿瘤医院张汝刚教授、解放军总医院周乃康教授、北京友谊医院王天佑教授、北京胸科医院许绍发教授、北京大学第一医院李简教授和北京医学会胸外科专业委员会副主任委员李辉教授用珍贵的历史资料和图片分别回顾了北京协和医院、中国医学科学院肿瘤医院、驻京部队系统医院、首都医科大学系统医院、北京大学系统医院和北京胸科医院的胸外科发展历史和北京医学会胸外科专业委员会的发展史。北京是我国胸外科的发源地，从1937年北京协和医院胸外科王大同教授完成的第一例肺切除手术到今天，北京胸外科已有70多年的历史，王大同、张纪正、吴英恺、张天民、范秉哲、辛育龄、黄国俊、黄孝迈、李曰民、古钰之、傅荛萁、孙衍庆、杨俊、朱大雷、蔡廉甫等胸外科老前辈为胸外科学科建设和人才培养、为北京胸外科事业的进步与发展做出了卓越的贡献。目前北京有三级甲等医院60余所，包括卫生部、中国医学科学院、首都医科大学、北京大学、清华大学、北京市属、国家各部委和驻京部队系统医院，共有区县二级以上医疗机构200余家，其中开展胸外科手术的医院有70余家，胸外科从业医生1 000多人。我国胸外科史上几乎所有的第一例手术均发生在北京，如第一例肺切除手术、第一例食管癌切除手术、第一例肺移植手术和第一例胸腔镜手术等。中华胸心血管外科学会和中国胸外科医师协会均设在北京。北京的胸外科技术水平代表了中国的胸外科技术水平。

首都医科大学宣武医院胸外科主任支修益教授就肺癌国际新分期和早期肺癌外科治疗新策略进行了专题演讲，介绍了2009年7月在美国旧金山召开的第十三届世界肺癌大会上公布的第七版国际肺癌TNM分期系统及其对早期肺癌外科治疗策略的影响；中国医学科学院肿瘤医院副院长赫捷教授介绍了新世纪中国食管癌流行病学状况与外科治疗新策略；北京协和医院胸外科主任李单青教授介绍了纵隔肿瘤尤其是胸腺肿瘤现代外科诊疗的新技术和新方法；中国人民解放军总医院胸外科主任初向阳教授就微创外科新技术和达芬奇机器人在胸外科领域的应用进行了演讲和手术演示，提出了单操作孔胸腔镜肺癌切除手术的新概念；北京大学肺癌专业学组组长张力健教授就肺癌规范化诊疗和卫生部即将颁布的肺癌临床诊疗路径进行了专题介绍，呼吁胸外科医生规范肺癌临床诊疗行为；卫生部北京中日友好医院胸外科主任刘德若教授介绍了肺减容手术治疗肺气肿的最新进展，特别是电视胸腔镜肺减容手术的技术要点；首都医科大学附属北京朝阳医院副院长候生才教授回顾了我国肺移植发展史和目前肺移植现状及进展，就积极推进和开展肺移植工作提出了很好的建设性意见；首都医科大学附属北京胸科医院院长许绍发教授就现代肺结核外科治疗策略和临床诊疗路径进行了专题演讲，明确指出我国肺结核的流行趋势不甚乐观、原发耐多药肺结核发病率逐年增高，目前仍有2%-5%的肺结核患者需要外科手术治疗；中国人民解放军空军总医院胸外科主任朱彦君教授在年会上报告了胸腔镜交感链切断术治疗手汗症的临床结果，初步的结果令人振奋。北京大学肿瘤医院胸外二科主任杨跃教授和首都医科大学宣武医院胸外科副主任张毅教授分别就非小细胞肺癌术前新辅助化疗、术后辅助化疗和分子靶向治疗专题进行了专题演讲。与会代表围绕会议演讲主题进行了热烈讨论。

各位特邀演讲专家的专题演讲中都突出一个“新”字，新思维、新技术、新方法、新进展是各位特邀专家演讲时听到的最多的词语。专题演讲涵盖了胸外科领域的各个专题，充分展示了北京地区胸外科作为全国的排头兵在胸外科各个领域的技术水平、学术成果和学科经营理念。2009年北京胸外科学术年会同期举办了北京地区胸外科护士长学术沙龙暨首届胸外科护理学术研讨会，共有来自北京地区各系统、各医院胸外科的70多名护士长和240余名胸外科护士出席了本届胸外科护理研讨会，就胸外科护理领域新技术、新进展进行了充分讨论。

